# Awareness and Knowledge of Sexually Transmitted Infections among Secondary School Adolescents in Ado Ekiti, South Western Nigeria

**DOI:** 10.1155/2015/260126

**Published:** 2015-08-09

**Authors:** E. O. Amu, P. T. Adegun

**Affiliations:** ^1^Department of Community Medicine, Ekiti State University Teaching Hospital, Ado Ekiti, Ekiti State 30001, Nigeria; ^2^Department of Surgery, Ekiti State University Teaching Hospital, Ado Ekiti, Ekiti State 30001, Nigeria

## Abstract

*Objective*. To determine the awareness and knowledge of sexually transmitted infections among adolescents in Ado, South Western Nigeria. *Methods*. The study was a descriptive cross-sectional design. Five hundred and fifty adolescents selected from public and private secondary schools in Ado Local Government Area of Ekiti State were recruited using a multistage sampling technique. *Results*. Four hundred and ninety-nine (92.4%) respondents had heard about sexually transmitted infections before, the three most important sources of information being electronic media (68.7%); teachers (68.1%); and print media (44.9%). Eighty percent of the respondents knew only one STI and the two most commonly mentioned ones were HIV/AIDS (78.0%) and gonorrhea (23.0%). More than 75% of the respondents knew the modes of transmission of STIs while some of them equally had misconceptions. The most important symptoms mentioned were weight loss (77.4%), painful micturition (68.9%), and genital ulcer (54.1%). On the whole, only 6.9% of the respondents had good knowledge of STIs; the rest had fair and poor knowledge. *Conclusion*. Secondary school adolescents in Ado Local Government Area have only a fair knowledge of sexually transmitted diseases. STI studies should be inculcated into the school curriculum and media publicity/enlightenment campaigns about them should be intensified.

## 1. Introduction

Sexually transmitted infections (STIs) are those diseases that are contracted mainly through sexual intercourse. They include curable ones like gonorrhea, syphilis, and chlamydia infection as well as incurable but modifiable ones like HIV, herpes simplex, human papilloma virus (HPV), and hepatitis B infections [1, 2].

Adolescents and young adults, aged 15–24 years, are more at risk for STIs than older adults. The World Health Organization estimates that 20% of persons living with HIV/AIDS are in their 20s and one out of twenty adolescents contract an STI each year [3]. Youths are more likely to practice unprotected sex, have multiple sexual partners, and have transgenerational and transactional sex. The cervical lining in female adolescents and young women makes them more predisposed to STIs. In addition, they may have problems getting the required information, services, and supplies they need to avoid STIs. They may also experience difficulties in accessing STI prevention services because they do not know where to find them, do not have transportation to get there, or cannot pay for the services. Even if they can obtain STI prevention services, they may not feel comfortable in places that are not youth friendly [4].

Untreated or poorly treated STIs are associated with a lot of complications. In males, gonorrhea as well as chlamydia trachomatis infection causes epididymitis which can result in infertility in the future. In addition, inflammatory urethral stricture may arise from poorly treated gonococcal urethritis in the future. This may lead to urinary retention and possibly chronic renal failure if not properly managed. For the females, pelvic inflammatory disease, dyspareunia, infertility, chronic pelvic pain, increased risk of ectopic pregnancies, abortions, stillbirths, and perinatal and neonatal morbidities can occur, jeopardizing their future reproductive competences [5].

Knowledge of STI and their complications is important for adequate prevention and treatment, as people who do not know the symptoms may fail to recognize their need and so may not seek help. Knowledge of other STIs apart from HIV/AIDS is low in the developing world [6–8].

Importantly, literatures on the awareness of STIs in Ekiti State are quite scanty if any. This study was conducted to determine the level of knowledge of adolescents in Ado Local Government Area of Ekiti State, Nigeria, about sexually transmitted infections, to identify their specific health educational needs and make appropriate recommendations to the Government and Ministry of Education.

## 2. Materials and Methods

### 2.1. Background to Study Area

Ado Local Government Area (LGA) is one of the sixteen LGAs in Ekiti State. It is made up of ten wards: Ado metropolis and the surrounding villages. These include Ago Aduloju and Aso Ayegunle. Ado LGA, a predominantly urban LGA, has as the headquarters Ado Ekiti which also doubles as the capital of Ekiti State. It is bounded in the North, West, South, and East by Ikere, Ifeoluwa, Ilawe, and Gbonyin LGAs, respectively. The land area is approximately 985 square kilometers.

According to the 2006 population census, the total number of people in Ado LGA is 129, 996 [9]. The people are predominantly Yorubas but other ethnic groups such as Igbos, Hausas, Isokos, Binis, and Ebiras collectively form a sizeable proportion of the population. The majority of the people are predominantly traders, artisans, and motor bike drivers; others are farmers and civil servants. There are many public and private primary and secondary schools and three tertiary institutions within Ado Local Government Area.

#### 2.1.1. Target Population

We targeted male and female secondary school students, in public and private secondary schools in Ado LGA, Ekiti State, Nigeria.

#### 2.1.2. Study Population

The study population was SS1–SS3 students attending public and private secondary schools in Ado LGA. In Nigeria, students spend 6 years in the primary and 6 years in secondary school. The first 3 years in the secondary school are referred to as Junior Secondary 1–3 or JS1–JS3. The latter 3 years are referred to as Senior Secondary 1–3 or SS1–SS3. On completion of the senior secondary school, students are meant to proceed to a university or another tertiary institution where they spend a minimum of 4–6 years depending on the course of study.

#### 2.1.3. Study Design

This was a prospective cross-sectional descriptive survey.

#### 2.1.4. Sample Size Determination

Sample size for the study was determined using the formula for calculating single proportions by Abramson and Gahlinger [10]. The total number of secondary school students in Ado LGA was above 10,000. Therefore the sample size formula

(1) $$\begin{array}{c} n=\frac{p\left(1-p\right)\times {Z_{\alpha }}^{2}}{d^{2}}, \end{array}$$

was used, where *n* is minimum sample size, *Z*_*α*_ is standard normal deviate, corresponding to 95% confidence level at which *Z* = 1.96 for a two tailed test, *p* is proportion in the target population estimated to have a particular characteristic (prevalence of STI from a previous study was 34%) [11] and *d* is degree of accuracy desired or maximum allowable difference from true proportion which was set at 5% (0.05); *n* = (0.34 × 0.66 × (1.96)^2^)/(0.05)^2^ = 345.

In order to make up for incompletely filled questionnaire, the number was increased to 500 but 550 respondents were interviewed.

#### 2.1.5. Sampling Technique

Multistage sampling technique was used for the study.

A complete list of all the private and public secondary schools in Ado Ekiti LGA was obtained from the Ministry of Education. Using systematic random sampling method, 3 private and 3 public secondary schools were chosen. The number of students chosen from each school was proportionate to the total population of students in the school. Simple random sampling (balloting) was used to select an arm from each of the class levels (SS1–SS3). The total sample size was shared among these chosen classes by proportionate allocation using the formula

(2) Class sample size=Total Class SizeTotal School Size (SS1 to SS3)×Total Sample Size.

This yielded the number to be drawn from each class. Simple random sampling technique was then to be used to draw out the number from each class using a table of random numbers.

#### 2.1.6. Data Collection Instrument and Methods

A pretested, self-administered questionnaire was used for data collection. The questionnaire elicited information on the sociodemographic characteristics of the respondents and their knowledge of STIs.

The questionnaire was pretested among students whose schools were not chosen as part of the study.

#### 2.1.7. Data Analysis

Data analysis was done using SPSS version 16. Univariate analysis (frequencies and percentages) was done. In determining the level of knowledge of each respondent about STI, a seventeen- (17-) point scale developed by the researcher was used. Question 9 with 4 stems on names of STIs known; question 10 with 7 stems on knowledge of modes of STI transmission, and question 11 with 6 stems on knowledge about symptoms of STI of the questionnaire were scored. Therefore, the total points obtainable by a respondent were seventeen (17). Each correct response was scored one mark and nonresponse or wrong response was scored zero mark. Those who scored six points or less (≤6) were considered as having poor knowledge; those who scored between seven and twelve (7–12) were considered as having fair knowledge, while those who scored between thirteen and seventeen (13–17) were considered as having good knowledge.

#### 2.1.8. Ethical Issues

The major ethical concern was that of confidentiality. The questionnaires were completed privately and anonymously. All records and relevant materials were stored in locked cabinets and accessed only by authorized personnel.

Ethical clearance was obtained from the Ethics and Research Committee of the University of Ado Ekiti Teaching Hospital. Permission to carry out the study was sought from the Ministry of Education and the Principals of the various schools. Written informed consent was also obtained from the participants.

## 3. Results

A total of 540 out of 550 questionnaires administered were correctly filled out and returned (response rate, 98.2%).

*Table 1*. The respondents were mainly aged between 10 and 19 years. Of these, 109 (20.2%) of them were aged between 10 and 14 years while 429 (79.4%) were aged between 15 and 19 years. The mean age was 15.7 ± 1.5 years. A total of 128 (23.7%) respondents were in SS1, 253 (46.9%) in SS2, and 159 (29.4%) in SS3 classes. Three hundred and twenty-seven (60.6%) of the respondents were females; 482 (89.3%) were from public schools; 511 (94.6%) were Christians and 468 (86.7%) belonged to Yoruba ethnic group.

*Table 2*. A total of 499 (92.4%) respondents were aware of sexually transmitted infections while 41 (7.6%) were not aware. The three major sources of information in decreasing order of importance were the radio and television 343 (68.7%); teachers 340 (68.1%); and newspapers 224 (44.9%).

As shown in Table 3, 80.2% of the respondents knew only one STI, and 3.7% mentioned sickle cell anaemia (a genetic disease) as an STI.

*Table 3*. A total of 433 (80.2%) respondents knew only one sexually transmitted infection while only 15 (2.8%) respondents knew four. The most popularly mentioned ones were HIV/AIDS 421 (78.0%) and gonorrhea 124 (23.0%). Twenty (3.7%) of the respondents incorrectly mentioned sickle cell anaemia as a sexually transmitted infection.

As shown in Table 4 more than a tenth (12.2%) to about a quarter (22.0%) of the respondents had misconceptions about the mode of transmission of sexually transmitted infections.

*Table 4*. The most popularly known modes of transmission were unprotected sex 473 (87.6%); sharing of infected sharps 446 (82.6%); and infected blood and blood products 395 (73.1%). There were equally misconceptions that STI can be transmitted by coughing/sneezing 119 (22.0%), sharing toilets 87 (16.1%), and sharing plates 66 (12.2%).

As shown in Table 5, only 48.3, 48.0, and 47.2% of the respondents could identify genital swelling, body rash, and genital discharge which were very common symptoms. This implies that more than 50% of the respondents could not identify these common symptoms of STI.

*Table 5*. The three most commonly known symptoms of STI were weight loss (77.4%); painful micturition (68.9%); and genital ulcer (54.1%).

*Table 6*. Overall, 103 (19.1%) respondents had poor knowledge and 400 (74.1%) had fair knowledge while 37 (6.9%) had good knowledge of sexually transmitted diseases.

## 4. Discussion

The study examined awareness and knowledge of sexually transmitted infections among adolescents in Ado Ekiti, South Western Nigeria. Nearly all the respondents were aware of sexually transmitted infections. This finding is consistent with that of a study conducted in Malaysia, in which 92% of the respondents reported awareness of STDs [12]. It is also similar but higher than that of a study conducted among Thai adolescents in which 9 out of ten respondents were aware of STDs [13]. It is also higher that of a study conducted in Northern Nigeria in which 67% of adolescents were aware of STIs [14]. Awareness about sexually transmitted infections in general has increased over the last three decades since the advent of HIV/AIDS due to the widespread publicity given to the disease. However, awareness about other STIs might not be encouraging.

The major sources of information were the radio and television (electronic media), teachers, and newspapers. This contrasts with reports of a study conducted among adolescents in North Western Nigeria in which the major sources of information were school lessons, mass media, and health magazines [14] and that conducted in Thailand in which the major sources of information were school, Internet, and hospital/clinic [13]. The fact that the electronic media are the major source of information is due to the fact that most people have access to transistor radios and adolescents especially have cell phones sets with in-built radios. These give them continuous access to the news. Teachers and schools are playing increasing roles in disseminating information about STIs. This is as a result of sexuality education which is being progressively incorporated into the school curriculum. Moreover, students are taught about STIs in subjects like Basic Science, Biology, and Home Economics.

In this study, only 3% of the respondents could mention four STIs while majority of them (eight out of ten) knew only one sexually transmitted infection which was HIV/AIDS. Gonorrhea was the next most popularly mentioned STI but it was only known to two out of ten of the respondents. Respondents who knew about herpes and syphilis were less than ten percent altogether while others like HPV infection, hepatitis B, and chlamydia were not mentioned at all. This finding is similar to that reported by studies conducted in Tanzania, North Central Nigeria Thailand, Germany, and Europe in general in which the most commonly known STI was also HIV/AIDS [15–19]. However, knowledge about other STIs like gonorrhea, syphilis, chlamydia, and HPV was much lower than that of HIV/AIDS, different from what was obtained in this study and also from one study to another.

These studies show that while widespread publicity has been given to HIV/AIDS, other STIs with severe complications and which also predispose to HIV/AIDS have been relatively ignored. It is imperative that awareness be created about these STIs as well.

About 4% of the respondents had the misconception that sickle cell anaemia is an STI. While this reveals the level of their ignorance about the cause of the disease, it can also fuel the existing stigmatization and discrimination against people with this genetic disorder. A misconception of this nature was not found in other peer-reviewed literature.

Majority of the respondents knew that STIs could be transmitted through unprotected sex, sharing of infected sharps and via infected blood/blood products. This is consistent with the reports of various other studies conducted within and outside the country though these studies focused mainly on HIV/AIDS [19]. In another study conducted among Thai university students, almost everyone knew that sexual intercourse was a route of transmission of STD [13]. In this study, the respondents' knowledge about the transmission of HIV/AIDS could have informed their choices of infected sharps and transfusion with infected blood/blood products since most of them could not even mention the STIs that were transmitted via these routes apart from HIV/AIDS.

There were misconceptions about routes of transmission as well. The respondents felt that STDs can be transmitted through coughing and sneezing 22%, by sharing toilets 16%, and by sharing plates 12%. This is similar to but higher than that reported among Thai students, about 8.7% of which felt that sharing clothes/things was a route of STD transmission. Misconceptions about STD transmission weaken the motivation to adopt safer sexual behaviour and strengthen stigmatization against people such that they may be discouraged from accessing healthcare services.

In this study, the three most commonly mentioned symptoms of STI were weight loss, painful micturition, and genital ulcer. This contrasts with that reported among Thai students in which the most commonly mentioned symptoms of STI were penile/vaginal discharge and genital itching [13]. It also contrasts with that of a study conducted among youths in North Central Nigeria in which the most popularly known symptoms of STIs were rash, painful urination, and painful intercourse [16]. The differences in symptom mentioned in these studies could be due to the STI type the adolescent was aware of. They could also be due to the nature of the questions asked. Whereas open-ended questions were used in this study, close-ended ones were used in the other studies with various options which could have allowed for guessing.

Overall, less than a tenth of the respondents had good knowledge; about three-quarters had fair knowledge, while approximately one-fifth had poor knowledge of sexually transmitted diseases. The majority of the respondents could only mention one STI and some even mentioned a genetic disease as an STI; about a quarter had misconceptions about the modes of transmission of STI, while some could not identify some common symptoms of STI.

## 5. Conclusion

The study concluded that secondary school adolescents in Ado Ekiti are mostly aware of sexually transmitted infections but lack in-depth knowledge about these diseases, their symptoms, and modes of transmission. Comprehensive health education about other sexually transmitted infections (apart from HIV/AIDS) should be inculcated into the secondary school curriculum. Media enlightenment campaigns about these diseases should also be emphasized.

## Figures and Tables

**Table 1:** Sociodemographic characteristics of the respondents.

Characteristics	Frequency	Percentage
Age		
10–14	109	20.2
15–19	429	79.4
20–24	2	0.4
Total	540	100.0
Sex		
Male	213	39.4
Female	327	60.6
Total	540	100.0
Ethnic group		
Yoruba	468	86.7
Igbo	47	8.7
Hausa	10	1.9
Others	15	2.8
Total	540	100.0
Class		
SSS 1	128	23.7
SSS 2	253	46.9
SSS 3	159	29.4
Total	540	100.0
School type		
Public	482	89.3
Private	58	10.7
Total	540	100.0
Religion		
Christianity	511	94.6
Islam	29	5.4
Total	540	100.0
Ethnicity		
Yoruba	468	86.7
Ibo	47	8.7
Hausa	10	1.9
Others	15	2.8
Total	540	100.0

**Table 2:** Awareness of sexually transmitted infections and sources of information.

Awareness of STI	Frequency (*n*) = 540	Percentage
Aware	499	92.4
Not aware	41	7.6
Total	540	100.0

Sources of information	Frequency^*∗*^ *n* = 499	Percentage

Radio/television	343	68.7
Teachers	340	68.1
Newspaper	224	44.9
Talks/seminars	213	42.7
Friends/relations	211	42.3
Hospital workers	192	38.5
Billboards/posters	147	29.5
Other means	33	6.6

^*∗*^There were multiple responses.

**Table 3:** Number and types of sexually transmitted infections known by respondents.

Number of sexually transmitted infections known	Frequency (*n* = 540)	Percentage
None	41	7.6
One	433	80.2
Two	51	9.4
Four	15	2.8
Total	540	100.0

Name of sexually transmitted infection	Frequency *n* = 499^*∗*^	Percentage

HIV/AIDS	421	78.0
Gonorrhea	124	23.0
Herpes simplex	35	6.5
Sickle cell anaemia	20	3.7
Syphilis	15	2.8

^*∗*^There were multiple responses.

**Table 4:** Modes of transmission and misconceptions about modes of transmission of sexually transmitted infections.

	Frequency^*∗*^	Percentage
Mode of transmission		
Unprotected sex	473	87.6
Needles and syringes	446	82.6
Blood and blood products	395	73.1
Mother to child	383	70.9
Misconceptions about modes of transmission		
Coughing/sneezing	119	22.0
Sharing toilets	87	16.1
Sharing plates	66	12.2

^*∗*^There were multiple responses.

**Table 5:** Knowledge of respondents about symptoms of sexually transmitted infections.

Symptoms	Frequency^*∗*^	Percentage
Weight loss	418	77.4
Painful micturition	372	68.9
Genital ulcer	292	54.1
Genital swelling	261	48.3
Body rash	259	48.0
Genital discharge	255	47.2
Other symptoms	27	5.0

^*∗*^There were multiple responses.

**Table 6:** Overall knowledge of sexually transmitted infections among the respondents.

Level of knowledge	Frequency	Percent
Poor knowledge	103	19.1
Fair knowledge	400	74.1
Good knowledge	37	6.9
Total	540	100.0

## Appendix

### A. Questionnaire on Sexually Transmitted Infection

*Identification*

Name of School
Class
Date

#### A.1. Sociodemographic Characteristics of Respondents

(1) Age (in years)
(2) Sex of respondent
  Male
  Female
(3) Religion
  Christianity
  Islam
  Others (specify).............................................
(4) Ethnic group
  Yoruba
  Igbo
  Hausa
  Others (specify).............................................
(5) Class
  SSS1...............
  SS2....................
  SS3......................
(6) Type of School
  Public
  Private

#### A.2. Knowledge of Sexually Transmitted Infections (STIs)

(7) Have you ever heard of sexually transmitted infections (STIs)?
  Yes
  No
(8) What is your source of information?
  TV/radio
  Newspaper
  Public talks/seminars
  Billboards/posters
  Hospital/health workers
  Teachers
  Friends/relations
  Others (specify)
(9) Do you know any sexually transmitted infection? Mention the ones that you know
(10) How do people contract sexually transmitted infections? Write either Yes/No/I do not know in front of the options given
  From needles and syringes
  Blood and blood products
  Sharing the same plate with infected person
  Unprotected sexual intercourse
  From mother to child
  From sharing the same toilet with an infected person
  Exposure to cough and sneeze from infected persons
(11) What are common complaints in people with STI? Write either Yes/No/I do not know in front of the options given
  Weight loss
  Burning pain when passing urine
  Discharge from genital area
  Wound/sore in the genital area
  Body rash
  Swelling/boil around the genitals
  Others (specify).............................................
